# Structured reporting of x-rays for atraumatic shoulder pain: advantages over free text?

**DOI:** 10.1186/s12880-018-0262-8

**Published:** 2018-07-03

**Authors:** Franziska Schöppe, Wieland H. Sommer, Florian Schmidutz, Dominik Pförringer, Marco Armbruster, Karolin J. Paprottka, Jessica L. V. Plum, Bastian O. Sabel, Felix G. Meinel, Nora N. Sommer

**Affiliations:** 1 Department of Radiology, University Hospital, LMU Munich, Marchioninistr. 15, 81377 Munich, Germany; 2 Department of Orthopaedic Surgery, University of Munich (LMU), Marchioninistr. 15, 81377 Munich, Germany; 3 BG Trauma Center, University of Tübingen, Schnarrenbergstrasse 95, 72076 Tübingen, Germany; 4 Department of Trauma Surgery, Klinikum rechts der Isar, Technical University of Munich, Ismaningerstrasse 22, 81675 Munich, Germany; 5 Department of Diagnostic and Interventional Radiology, Rostock University Medical Center, Ernst-Heydemann-Str. 6, 18057 Rostock, Germany

**Keywords:** Shoulder pain, X-rays, Decision trees, Quality improvement, Clinical decision-making, Structured reporting

## Abstract

**Background:**

To analyse structured and free text reports of shoulder X-ray examinations evaluating the quality of reports and potential contributions to clinical decision-making.

**Methods:**

We acquired both standard free text and structured reports of 31 patients with a painful shoulder without history of previous trauma who received X-ray exams. A template was created for the structured report based on the template ID 0000154 (Shoulder X-ray) from radreport.org using online software with clickable decision trees with concomitant generation of structured semantic reports. All reports were evaluated regarding overall quality and key features: content, information extraction and clinical relevance.

**Results:**

Two experienced orthopaedic surgeons reviewed and rated structured and free text reports of 31 patients independently. The structured reports achieved significantly higher median ratings in all key features evaluated (*P* < 0.001), including facilitation of information extraction (*P* < 0.001) and better contribution to subsequent clinical decision-making (*P* < 0.001). The overall quality of structured reports was significantly higher than in free text report (*P* < 0.001).

**Conclusions:**

A comprehensive structured template may be a useful tool to assist in clinical decision-making and is, thus, recommended for the reporting of degenerative changes regarding X-ray examinations of the shoulder.

**Electronic supplementary material:**

The online version of this article (10.1186/s12880-018-0262-8) contains supplementary material, which is available to authorized users.

## Background

Clear and unambiguous X-ray reports are a prerequisite for interdisciplinary patient management and enable the radiologist to add value to the clinical process of proper patient care. Comprehensive and precise reports are crucial to avoid misunderstandings and miscommunication between radiologists and referring physicians [1] which might even affect patient management negatively [2].

There have been statements and initiatives by several radiological societies on the potential and the preferable use of structured reports (SR) and standardized terminology such as RadLex, the reporting initiative of the Radiological Society of Northern America (RSNA) [3–5] and the reporting guidelines issued by the European Society of Radiology [6].

There has been increasing evidence more recently that SR are preferred over free text reports (FTR) by both radiologists and referring physicians [7–13]. Several previous studies evaluating the use of SR in different imaging modalities, including radiography, sonography, computed tomography (CT) and magnetic resonance imaging (MRI) [12–16], provide evidence on the potential of SR with respect to completeness, accuracy, perceptions and satisfaction of the referring clinicians. Moreover, a survey among North American radiologists from academic teaching hospitals found out that approximately half of them use SR at least for some reports [17]. Thus, the existing studies point to the great potential of the implementation of SR in clinical settings. However, there are also some possible disadvantages of SR such as the risk of over-simplification, distraction by the introduction of additional SR tools, cumbersome decision trees and the possibility of reduced detection rates [18–20]. Furthermore, the implementation of SR might lead to prolonged reporting times, especially in the beginning and among radiologists who are used to report by free speech dictation or free text entry [21].

Consequently, the use of SR up to now has not yet been widely established as clear evidence of the superiority of SR over FTR is lacking. Therefore, studies comparing template-based SR with FTR might provide further evidence for the advantage and potential of SR and facilitate the implementation process.

Chronic shoulder pain is common in the elderly population and X-ray examination is the first step in the diagnostic imaging process. To the best of our knowledge, no study has yet evaluated template-based SR compared to conventional FTR for the radiographic examination of degenerative diseases of the shoulder. Therefore, the aim of the current study was to compare the quality of SR and FTR regarding the radiographic examination of degenerative diseases of the shoulder.

## Methods

### Patient selection and study design

After approval by the Ethics Committee of our institution a retrospective search was performed in our database of radiologic reports’ containing all exams from May 1, 2013 to March 1, 2016. Written informed consent was waived by the Ethics Committee as data were de-identified and analysed anonymously. We identified all radiographic shoulder exams which had been acquired for clinical reasons at our hospital. Images and patients were included if they had been presenting with a painful shoulder without history of a previous trauma. Patients were excluded if they had undergone shoulder arthroplasty or were suffering from a bone tumour. Images were acquired in at least two planes (15° anterior-posterior and lateral) for all patients.

### Sample size calculations

As previously described by another study on structured reporting [12] we also based our sample size calculations on the anticipated effect size measured as the increase of the proportion of reports with high/very high overall quality ratings. Assuming a baseline proportion of 50% of the FTR receiving high/very high quality ratings and 85% of the SR with high/very high quality ratings our estimated effect size was 35%. To be able to detect this difference with a power of 80% at a level of significance of α = 0.05 the minimum required sample size would be *N* = 54 reports (27 for each report type). To account for a possible overestimation of the effect we adjusted the sample size by adding 15% which lead to the final sample size of *N* = 62 (31 per group).

Therefore, the first 31 consecutive exams fulfilling the inclusion and exclusion criteria mentioned above were used for further evaluation.

### Radiologic reports

**Fig. 1:**
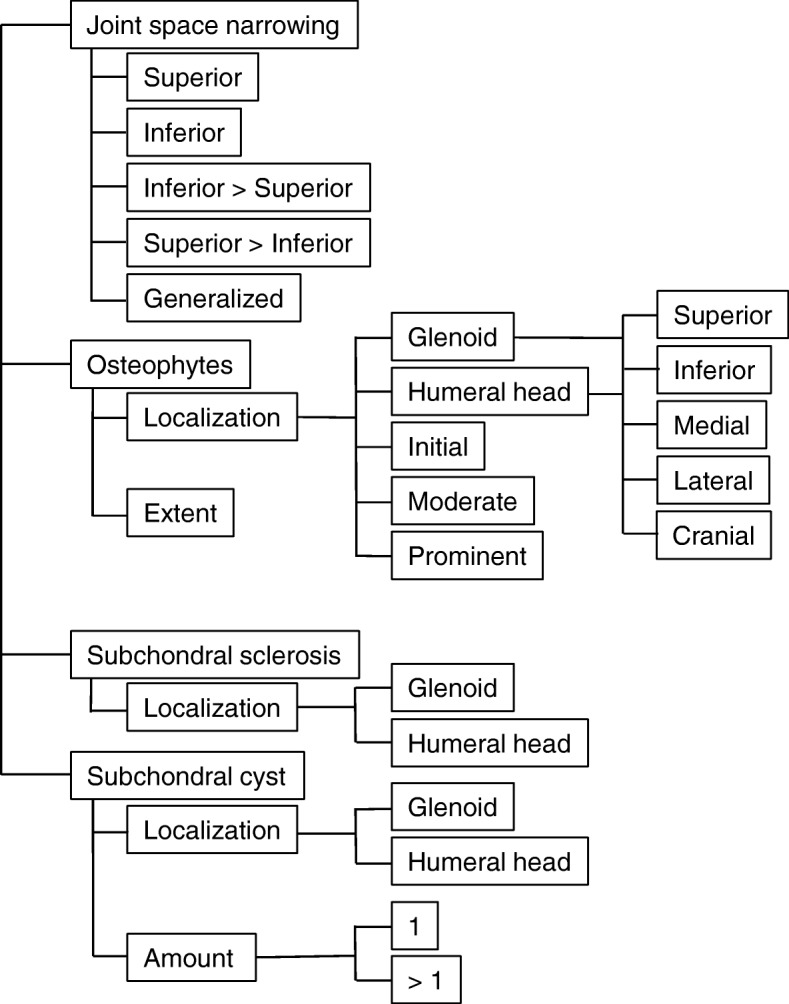
Decision tree (extract). Exemplary section of the decision tree for the evaluation of degenerative changes of the glenohumeral joint due to osteoarthritis (slightly modified for illustration purposes)

The standard FTR was compared to SR for each patient. The FTR were taken from the daily routine reports created by using a standard speech recognition software (Philips SpeechMagic 6.1, Build 543 SP1 (7/2007), Philips Speech Recognition Systems GmbH). The SR were generated using a structured template created previously. This template of degenerative changes of the shoulder was based on the RSNA Radiology Reporting Templates Shoulder Xray, template ID 0000154 from radreport.org [22]. We created the SR using online software (Smart Reporting, www.smart-radiology.com, a not-for-profit company) with clickable decision trees and the concomitant generation of a semantic SR. The decision tree included the report section with elements such as previous exams, foreign material and details on anatomical structures, including degenerative changes. More detailed information could be entered for the glenohumeral joint and the acromioclavicular joint by defining the presence and the extent of radiographic signs of osteoarthritis such as joint space narrowing, osteophytes, subchondral sclerosis and subchondral cysts. Figure 1 shows exemplarily the elements used for the evaluation of degenerative changes of the glenohumeral joint. Furthermore, possible deformities of the humeral head could be selected in the section on articulation of the humeral head (not shown).

Following the selection of the radiographic features mentioned above the severity of osteoarthritis could be classified according to the Kellgren and Lawrence classification as grade 0 (no radiographic sign of osteoarthritis) to IV (including joint space narrowing, subchondral sclerosis and cysts, osteophytes and deformity) [23] in the impression section.

Selectable key elements for the acromion and the subacromial space were narrowing of the subacromial space, calcific tendinitis, acromion type and the presence of subacromial osteophytes. Additionally, the template contained elements on general information such as fractures, bone structure and density, as well as elements concerning the surrounding soft tissues. To avoid unwanted rigidity of the template, we included a free text element for any additional information not covered by the clickable decision tree.

Based on our template a radiologist with 2 years of experience in musculoskeletal radiology re-read all the shoulder X-ray exams and created SR. The template-based text output was exported to the clipboard by one click and immediately afterwards pasted into a text file. The original FTR was not used when creating the SR. All reports were anonymized using a unique identification number. Bold font was used for emphasis of pathological findings.

### Evaluation of the reports

The FTR and SR were randomly rearranged based on a computer-generated randomization scheme for further analysis. We created a questionnaire for the evaluation of the reports using an online-based survey tool (LimeSurvey, http://www.limesurvey.org [24]). The questionnaire consisted of four different parts: (A) content-related questions (three items), (B) questions about structure, layout and comprehensiveness of the reports (three items), (C) clinical consequence of the report as perceived by the referring physician (two items) and (D) overall quality (one item) (see Table 1). We used a 10-point Likert scale (0 = I do not agree, 10 = I agree) for all but the last question on overall quality. A 5-point Likert scale was employed (0 = insufficient, 1 = poor, 2 = acceptable, 4 = good, 5 = very good) for overall quality.

**Table 1:** Parts and items of the questionnaire for the evaluation of reports on X-ray exams of the shoulder

Part	Item
A – content related	1. The report contains detailed information whether and to what extent signs of osteoarthritis are present.
	2. The report contains information on the subacromial space/acromion (e.g. width, calcific tendinitis, acromion type, etc.).
	3. The report contains additional relevant information.
B – structure, layout and comprehensiveness	1. The structure/highlighting of the elements is helpful for information extraction.
	2. The extent of the report is appropriate.
	3. The linguistic comprehensibility of the report is good.
C – Clinical consequences	1. The clinical question is answered in the report.
	2. Based on the report, a decision on further clinical management of the patient (e.g. therapy, additional diagnostic tests required) can be made without the need for further consultation of the reporting radiologist.
D – Overall quality	1. How do you rate the overall quality of the report?

The anonymized reports were evaluated independently and separately in a randomized order of all reports by two experienced orthopaedic surgeons (experience in shoulder X-ray exams of five and seven years, respectively). The questionnaire had to be answered correspondingly immediately after reading the report. All reports were evaluated by the referring physicians in one session. The evaluating orthopaedic surgeons were only given the written reports and did not see the imaging exams themselves.

### Statistical analysis

The results of the 10-point Likert scale ratings were considered as paired continuous data for the statistical analysis of the results. The ratings of each item were compared between FTR and SR using the Wilcoxon-Mann-Whitney U-Test. The overall quality ratings were considered to be categorical and were, thus, analysed using the McNemars test to compare the ratings between the two groups. The statistical analysis was performed using IBM® SPSS® Version 20.

## Results

We included X-ray exams of the shoulders of 31 patients. There was an anonymized FTR and SR (*N* = 62 reports in total) for each patient that were evaluated by two orthopaedic surgeons using the online survey tool (*N* = 124 completed questionnaires). The results of each item are reported as medians and interquartile ranges as the ratings are not normally distributed within the groups.

### Satisfaction with content

This section focused on the satisfaction with the content of the report and evaluated the presence and completeness of information on degenerative changes, e.g. osteoarthritis and calcific tendinitis. The SR were rated significantly higher than FR regarding the content of detailed information about osteoarthritis (*P* < 0.001, see Table 2), for information on the subacromial space and the acromion (*P* > 0.001), and for additional relevant information (*P* < 0.001).

**Table 2:** Overview of the ratings for structured reports (SR) vs. free text reports (FTR) for the items of the questionnaire with a 10-point Likert scale (0 = I do not agree, 10 = I agree)

Part	Item	Median rating (Interquartile range)		Wilcoxon-Mann-Whitney U
		SR	FTR	
A – content related	1. The report contains detailed information whether and to what extent signs of osteoarthritis are present.	10.0 (10.0–10.0)	5.0 (2.0–7.0)	*P* < 0.001
	2. The report contains information on the subacromial space/acromion (e.g. width, calcific tendinitis, acromion type etc.).	10.0 (10.0–10.0)	5.0 (2.0–8.0)	*P* < 0.001
	3. The report contains additional relevant information.	10.0 (10.0–10.0)	4.0 (2.0–8.0)	*P* < 0.001
B – structure, layout and comprehensiveness	1. The structure/highlighting of the elements is helpful for the information extraction.	10.0 (10.0–10.0)	2.5 (1.0–4.0)	*P* < 0.001
	2. The extent of the report is appropriate.	10.0 (10.0–10.0)	3.0 (1.0–8.0)	*P* < 0.001
	3. The linguistic comprehensibility of the report is good.	10.0 (10.0–10.0)	6.0 (2.0–9.0)	*P* < 0.001
C – Clinical consequence	1. The clinical question is answered in the report.	10.0 (10.0–10.0)	5.0 (3.0–8.0)	*P* < 0.001
	2. Based on the report a decision on further clinical management of the patient (e.g. therapy, additional diagnostic tests required) can be made without the need of further consultation of the reporting radiologist.	10.0 (10.0–10.0)	4.0 (2.0–8.0)	*P* < 0.001

### Satisfaction with structure, highlighting and comprehensibility

The greatest difference between the average ratings was observed for the section focusing on the value of structure and highlighting of elements for information extraction, with SR receiving higher ratings than FTR (*P* < 0.001). The extent of the SR was found to be more appropriate than that of the FTR (*P* < 0.001). The comprehensibility was also rated significantly higher for SR compared to FTR (*P* < 0.001).

### Satisfaction with impact on clinical decision-making

The SR addressed the clinical question of the referring physician better than FTR (*P* < 0.001). The contribution to the subsequent clinical decision-making without the need for additional consultation of the reporting radiologist was rated significantly higher for the SR compared to the FTR (*P* < 0.001).

### Overall quality ratings

**Fig. 2:**
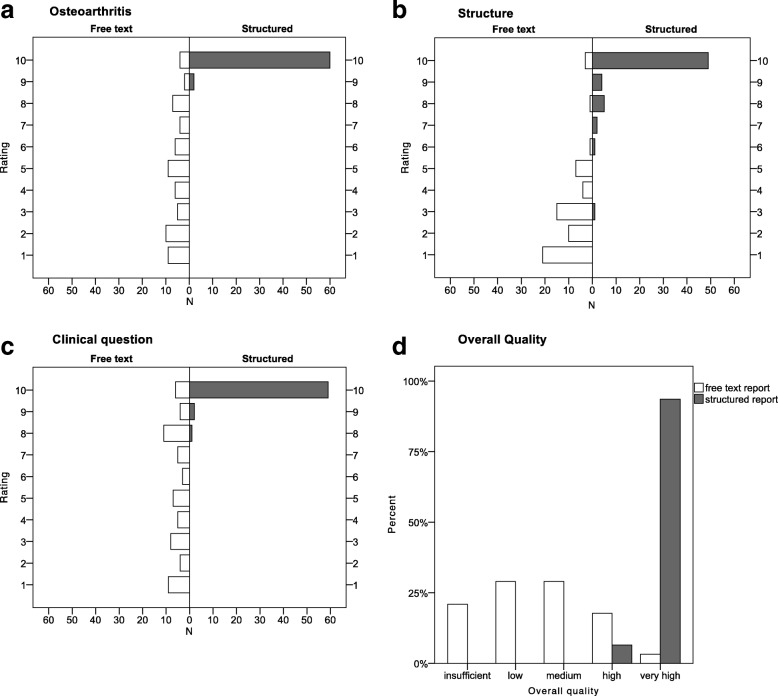
Ratings by report type for selected questionnaire items. **a**-**c** show exemplary histograms of the ratings on a 10-point Likert scale (0 = I do not agree, 10 = I agree) for the items on osteoarthritis (**a**), structure (**b**) and the report addressing the clinical question (**c**) for FTR (*N* = 62 ratings; white bars on the left) and SR (N = 62 ratings; grey bars on the right); D shows the percentage of the ratings of overall quality by each category for FTR and SR

The SR also achieved significantly higher overall quality ratings compared to the FTR (*P* < 0.001) (Fig. 2). The overall quality for all SR (*N* = 62 ratings, 100%) was rated either as “high” (*N* = 4, 6.5%) or “very high” (*N* = 58, 93.5%), whereas for the FTR (N = 62 ratings, 100%) only *N* = 13 (20.9%) of the FTR received either a “high” or “very high” quality rating. The two most frequent overall quality categories were “medium” and “low” (in each of these categories *N* = 18 reports (29%)).

A total of 21% (N = 13) of the FTR were even considered to be of insufficient quality.

Overall, the SR were rated significantly higher than the FTR for all nine items of the questionnaire.

## Discussion

In our study, SR received significantly better ratings for all items compared to FTR. The SR were considered to be either of high or very high quality whereas FTR obtained significantly lower ratings for the overall quality of the reports. Furthermore, there was a great heterogeneity among the FTR, as indicated by the large interquartile range whereas the SR had consistently high ratings with very little variation.

As far as we are aware, this is the first study evaluating template-based SR compared to FTR in patients receiving an X-ray examination for the assessment of painful degenerative shoulder joint alterations. The results of a higher homogeneity among SR are in line with a previous study evaluating SR in CT examinations of patients suspected of pancreatic cancer [13]. Another study which aimed to compare the quality of SR in staging of rectal cancer with MRI to FTR displayed an improved quality when using SR [12]. According to our results, the template-based SR for the X-ray examination of shoulder pain also lead to an improved overall quality as perceived by the referring surgeons. As the ratings of SR and FTR differed significantly for all items, the results clearly implicate that the referring physicians who rated the reports in our study prefer SR for the radiographic evaluation of atraumatic shoulder pain. This is in line with previous studies that underline the preference for SR by referring physicians [9, 15].

Nevertheless, it cannot be simply assumed that the use of SR leads uniformly to an increased completeness, as illustrated by a study by Johnson et al.: The authors did not find a significant difference regarding completeness and accuracy scores for radiology residents’ SR and FTR of cranial MRI studies in patients suspected of having a stroke [10]. Therefore, it is crucial to validate each newly designed structured template.

Furthermore, the SR were rated as being more complete, especially with regard to content-related items. As this is crucial for further patient management, our template might assist with an improved radiologic report to enhance the clinical decision-making process and treatment of patients. Our results on the improved completeness of reports when using SR compared to FTR is in accordance with previous findings from a study evaluating SR versus FTR of chest X-ray exams and the study on reports of pancreatic cancer CT exams mentioned above [12, 13, 16]. However, some radiologists argue that an improved clarity might be at the expense of a comprehensive report that is able to adequately address the complexity of imaging findings [19].

A further advantage of providing SR is the simplified extraction of relevant information. Our template follows a standardized structure and order of the elements included, designed to make the reports comparable to each other with a high recognition value. Thus, one could speculate that, after a short implementation phase of getting used to the transition from FTR to SR, the referring physician would able to identify the relevant findings, resulting in time-saving without missing relevant information. Another point that deserves attention is the potential advantage for less experienced radiology residents for whom the templates of SR may serve as a kind of checklist, which has been shown to improve patient care in various medical fields [25–28]. Whether the use of a clickable decision tree in SR might even lead to a decrease of reporting times for the radiologist, especially those more experienced, is currently being debated controversially. One study found short reporting times [29], whereas others found prolonged reporting times [10, 18] underlining the need for further studies.

Moreover, the use of an online-based template, such as in our case, can be seen an important prerequisite for vendor-neutral use by copying and pasting the SR generated into the current established radiologic reporting software. However, the use of an online-based template is not a prerequisite for the generation of SR and should only be seen as one means amongst others. To allow a smooth integration of structured reporting into the daily clinical routine, an integration of SR tools in the existing reporting software would be favourable.

Our study has a few limitations due to the study design. One limitation is that the reports were only evaluated by two orthopaedic surgeons (representing only one subspeciality) from two different university hospitals with a similar level of experience. It is, therefore not possible to generalize our findings towards a preference for SR of radiographic exams of the shoulder for all referring physicians. A review of the reports by a more varied group of providers would have been beneficial. There might be differences in the preference of SR compared to FTR depending on the specialty, the clinical setting (e.g. outpatient care) and level of expertise of the referring physician. Although the referring physicians evaluated all cases in one session in a randomized order, we do not believe that this possibly influenced the ratings, since only the report examinations were evaluated.

Secondly, we created a template that was tailored for patients with atraumatic shoulder pain who commonly present at our MSK outpatient clinic. Our aim was to create a template that is easy to use and to avoid that using the template becomes a cumbersome process by including several sublevels to accommodate all possible findings in X-rays of the shoulder. However, it is likely that the use of a more generalized template would further improve its potential for use in clinical practice. While our template is tailored for the evaluation of atraumatic shoulder pain it is not limited to patients with those complaints only and allows to report additional pathologies such as fractures. If there is no according clickable element predefined, the radiologist always has the possibility to make a free text entry. It has previously been pointed out that the introduction of several specialized templates might distract radiologist from the actual reporting [18]. On the other hand, there is evidence that specific templates (e.g. CT abdomen for suspected pancreatic cancer [13]) lead to a higher completeness of reports. We hypothesize that the solution may be a compromise between decision trees with many sublevels which cover a broad spectrum and tailored templates like the one we evaluated in our present study. Feasibility studies on the implementation of such templates into clinical routine will be necessary to further evaluate this aspect.

Furthermore, there is inherent bias in our study as we compared FTR acquired during clinical routine to SR that were generated in a research setting without time constraints. It is possible that a study that compared FTR and SR that were both generated in a research setting would reveal better ratings for FTR. The effect size when comparing FTR to SR in clinical routine is likely to be smaller than the one in our study. Nevertheless, we believe that our study can generate preliminary evidence on the quality of such templated-based SR. Such findings are crucial to justify the introduction of SR in radiological departments to test the feasibility in daily routine.

Keeping in mind the retrospective design of our study, we did not test the feasibility of implementing our template in clinical routine reporting. This aspect could be addressed in a prospective study. Future studies should also evaluate if and to what extend the introduction of SR into the clinical workflow confirms the concerns about potentially increased reporting times and the risk of distraction by using an additional structured reporting tool [20, 21].

## Conclusion

In conclusion, our study has shown that the generation of template-based SR resulted in higher completeness and overall quality of radiologic reports on X-ray exams of patients with atraumatic shoulder pain. When compared to conventional free text reports template-based structured reports on X-ray exams of patients with atraumatic shoulder pain have the potential to provide more complete reports, to facilitate information extraction and to lead to improved overall report quality. Thus, structured reporting appears to be a promising tool to enhance interdisciplinary communication and, thus, even might improve patient management. The clear preference of the referring surgeons for structured reports on X-ray exams of patients with atraumatic shoulder pain further supports the growing evidence that SR can improve current reporting practices.

## Keypoints

- Structured reports on shoulder X-ray exams lead to overall improved report quality.
- Reports generated by using clickable decision trees are more complete and accurate.
- Template-based reports add more value to clinical decision-making than free text reports.

## Additional file

Additional file 1: **Table S1.** Raw data of report ratings by referring physicians. (XLS 43 kb)

## Abbreviations

FTR: Free text reports
SR: Structured reports
